# Multicultural Efficacy Beliefs in Higher Education: Examining University Instructors’ Burnout and Mental Well-Being

**DOI:** 10.1177/00332941241253599

**Published:** 2024-05-13

**Authors:** Saghar Chahar Mahali, Phillip R. Sevigny, Shadi Beshai

**Affiliations:** Department of Psychology, 3653 University of Guelph, Guelph, ON, Canada; Department of Educational Psychology, 3158 University of Alberta, Edmonton, AB, Canada; Department of Psychology, 6846 University of Regina, Regina, SK, Canada

**Keywords:** Burnout, mental well-being, multicultural efficacy, higher education, diversity

## Abstract

Canadian universities are experiencing a dramatic increase in enrollment of students from diverse backgrounds. Evidence suggests many educators are not prepared to teach in multicultural contexts. Educators’ lack of preparedness to teach in such contexts may lead them to develop burnout, which can negatively impact their mental and professional well-being. However, self-efficacy beliefs may buffer against job burnout and promote mental well-being. Hence, multicultural efficacy is an important factor for teaching in multicultural settings. In this study, we examined the relationships of multicultural efficacy with university instructors’ burnout and mental well-being. A total of 158 faculty and sessional instructors were recruited from four prominent higher education institutions in Canada. The results revealed that multicultural efficacy was significantly related to the Personal Accomplishment facet of burnout and mental well-being, even after controlling variance accounted for by demographics, job-related characteristics, teaching self-efficacy, and colour-blind racial attitudes. These findings indicate that domain specific multicultural efficacy and general teaching self-efficacy are distinct constructs. Further, findings may inform the development of training opportunities and diversity-related workshops to enhance university instructors’ awareness of diversity, social justice issues, and multicultural efficacy to better equip them for instruction in multicultural classrooms.

## Introduction

Evidence suggests that burnout is prevalent among school teachers (e.g., García-Carmona et al., 2019) and university instructors (e.g., Fernández-Suárez et al., 2021). These unmitigated levels of burnout have important ramifications (Khan et al., 2019). Burned out educators experience low job satisfaction (Smetackova et al., 2019) and are more likely to have turnover intentions (Madigan & Kim, 2021). Fortunately, self-efficacy beliefs, known as evaluations of personal capabilities to perform a course of action successfully (Bandura, 1997), may buffer against job burnout (Aloe et al., 2014). Multicultural efficacy is a specific form of self-efficacy beliefs (Nadelson et al., 2012) and is defined as the teachers’ confidence in their ability to instruct in multicultural contexts effectively (Guyton & Wesche, 2005). These specific efficacy beliefs are noteworthy, as the proportion of students from diverse backgrounds enrolled in Western post-secondary institutions is increasing steadily. For instance, Canada witnessed a 68% increase in the proportion of international students from 2014 to 2018 (Government of Canada, 2019).

Students from diverse backgrounds encounter several challenges in the academic settings. For example, they may not be accustomed to the abroad educational system and have different educational experiences. Such students may face challenges to participate in classroom discussions, pose questions in a group setting, attend professors’ office hours, or reach out to them to seek clarity (Sanger, 2020). Scholars have also demonstrated unfamiliarity with teaching practices and uncertainty with criteria and structure of evaluation methods among Chinese undergraduate students in the United States (U.S.; Heng, 2018). Additionally, some international students may unintentionally engage in plagiarism due to cultural differences (e.g., the belief that reproducing an author’s original words verbatim is “a sign of respect”), unfamiliarity with proper citation regulations, and insufficient understanding of the significance of academic honesty (Fatemi & Saito, 2020, p. 1309). Addressing plagiarism can be “burdensome and difficult” for university instructors (Vehviläinen et al., 2018, p. 5). Furthermore, assisting these students to overcome the risk of plagiarism can involve some additional work for instructors, such as offering “extra consultations and exercises” to teach them appropriate ways of referencing (Adhikari, 2018, p. 385).

Therefore, to assist diverse students in their academic endeavour, university educators need to adjust their pedagogical and assessment approaches. For example, university educators can include various activities and instructional approaches (e.g., using “visual aids” or interactive technologies in lectures, activities conducted in small groups or pairs) to meet the needs of students with different learning styles (Sanger, 2020, p. 47). Various methods of evaluation can also be incorporated, which can include assignments using conventional (e.g., “analytic essays”) or other alternative modes of writing (e.g., “journalistic”), individual or group oral presentations, to name a few (p. 51). According to Sanger (2020), demonstrating awareness of diverse backgrounds of students through the “select[ion] of topics, author, assignments, and activities” can instill feelings of belongingness among students, which, in turn, “promote student engagement and learning” (p. 33).

Diversification of the course material is another suggested strategy to enhance the sense of belonging among diverse students and facilitate their learning (Sanger, 2020). One approach in multicultural teaching is the inclusion of topics and materials that recognize diverse perspectives and experiences (Khaja et al., 2010). The incorporation of such information can be challenging. In this vein, research conducted among university professors in a midwestern university revealed that some respondents considered this approach time consuming and effortful (Khaja et al., 2010). The study also indicated that several university instructors teaching science, statistics, and mathematics believed that multicultural teaching was not relevant to their courses and viewed basic science as culturally neutral and, therefore, did not dedicate time to it (Khaja et al., 2010). Similarly, in a study conducted among faculty members at a midwestern university, Bigatti et al. (2012) found that a small proportion of respondents held negative views towards multicultural teaching. The benefits of incorporating multicultural approaches in their course content was not recognized by some faculty. In this vein, a faculty mentioned that “[p]resenting material from differing points of view is very often not desirable when teaching a hard science - the facts are the facts and not open to interpretation based on your cultural group” (p. 85). Accordingly, it can be stated that diversification of course content can pose more challenges for instructors teaching social science courses (e.g., sociology) compared to those in the fields of natural sciences, as the latter group may not recognize its relevance. Despite this, “every course has several dimensions that can be considered in planning for change: content, instructional strategies and activities, assessment strategies, and classroom dynamics, including how instructors respond to student comments” (Kitano, 1997, p. 19).

Extant evidence suggests that many educators have low baseline multicultural self-efficacy. For example, the results of the Teaching and Learning International Survey (TALIS) revealed that, in most countries taking part in the study, the majority of teachers (i.e., more than 50%) lacked preparedness for instruction in multicultural classrooms (Organization for Economic Cooperation & Development, 2019). Educators may not receive sufficient training for providing instruction in multicultural settings (e.g., Dusi et al., 2017) despite the challenging nature of such contexts. Instructors may be hesitant to encourage individualistic thinking when students come from educational backgrounds that may not emphasize or promote such an approach (Witte et al., 2003). While this evidence is suggestive, to our knowledge, no studies to date have examined the direct associations of multicultural self-efficacy with burnout and well-being among post-secondary educators. Given that universities in the Western world are becoming more pluralistic, elucidating the links between multicultural efficacy, burnout, and mental well-being may aid in developing programs that specifically target such efficacy beliefs among university instructors.

The high demands of teaching are associated with high levels of work-related stress which over time may lead to burnout (Maslach et al., 1996; Palmer et al., 2015). Burnout is comprised of three components: Emotional Exhaustion (i.e., the depletion of one’s mental and physical energy), Depersonalization (i.e., having negative feelings and attitudes towards people that one is interacting with), and reduced Personal Accomplishment (i.e., one’s negative evaluation of his or her job performance; Maslach et al., 1996). Burnout appears to be widespread among post-secondary educators. In a systematic review, researchers found elevated levels of burnout and stress among university academics (Urbina-Garcia, 2020). Another systematic review of 12 studies which included a total sample of 2841 university academics reported a 37% prevalence rate of burnout among them (Fernández-Suárez et al., 2021). This widespread burnout among university educators has broad implications. For example, the review of the literature, which included a sample of 9110 faculty members, has revealed that burnout is related to a reduced sense of occupational satisfaction, poor psychological and physical health, withdrawal, and intention to quit among this population” (Sabagh et al., 2018).”

### Mental Well-Being and Self-Efficacy in Higher Education

As a result of high burnout and stress levels, educators in higher education often report reduced mental well-being and heightened distress. Shen et al. (2014) found depressive symptoms rates of 58.9% in their total sample of 1210 Chinese university instructors. Kataoka et al. (2017) found 10.2% of their sample of 342 Japanese university instructors were at high risk for depression. Similarly, a study of 1470 academics in Canada revealed high levels of psychological distress among 13% of them, which appeared to be more elevated than that of other Canadians in white-collar occupations (Catano et al., 2010). While the picture painted of increased burnout and reduced well-being among educators is grim, fortunately there are several known buffers to burnout and contributors to improved well-being. Self-efficacy beliefs enable individuals to be in control of their actions (Schwarzer et al., 1999). Bandura (1997) defines self-efficacy beliefs as individuals’ assessments of their capabilities in executing a particular task effectively. When encountering challenging situations, individuals with high self-efficacy demonstrate greater effort and persist longer in executing a specific task compared to those with low self-efficacy. Bandura (1997) asserted that self-efficacy beliefs are important in fostering one’s well-being, reducing stress, and decreasing the likelihood of developing depression.

Self-efficacy in teaching is a specific application of Bandura’s (1997) theory. Teacher self-efficacy can be described as “a teacher’s individual beliefs in their capabilities to perform specific teaching tasks at a specified level of quality in a specified situation” (Dellinger et al., 2008, p. 752). Highly self-efficacious teachers have more enthusiasm and confidence about teaching and utilize various methods of instruction (Allinder, 1994). Teachers’ self-efficacy beliefs are linked to student achievement and motivation (Mojavezi & Tamiz, 2012). In addition to their positive influence on students, self-efficacy beliefs can contribute to educators’ well-being. Highly self-efficacious teachers are less likely to experience burnout (Capone et al., 2019), depression, and anxiety and tend to be more contented and enthusiastic (Huang et al., 2019). In their meta-analysis, Aloe et al. (2014) found that teachers who harboured higher levels of classroom management self-efficacy had lower levels of burnout. Accordingly, self-efficacy beliefs seem to have a buffering effect against the feelings of stress and burnout (Aloe et al., 2014). While most studies on self-efficacy beliefs to date tend to focus on school teachers (as noted by Noben et al., 2021), similar patterns of results have been observed in the context of post-secondary education. For example**,** Han et al. (2021) found that teaching efficacy among a sample of 2758 university faculty in China was positively related to occupational engagement and satisfaction. Similarly, utilizing a sample of 251 university instructors in Germany along with the reports of 9241 students, Daumiller et al. (2016) found that university instructors’ teaching self-efficacy was positively associated with students’ learning and their overall evaluation of instructors’ teaching quality.

Larke (2013) has defined culturally responsive teaching as “an instructional pedagogical strategy whose main purpose is to address the needs of all students” (p. 39). According to Larke (2013), the creation of a course that incorporates the principles of this pedagogical approach is contingent upon instructors’ cultivating foundational understanding and awareness of issues surrounding diversity. Similarly, Quaye and Harper (2007) have mentioned that the creation of an effective inclusive learning environment for students requires university instructors to actively engage in critical self-assessment with respect to their “assumptions, biases, and knowledge insufficiencies” (p. 38). Further, university educators should consider it their responsibility to acquire the essential knowledge and skills needed to incorporate diversity into their teaching methods (Quaye & Harper, 2007).

Given that Canadian universities are experiencing a dramatic increase in the number of students from diverse backgrounds, it is important to assess educators’ self-efficacy beliefs in the context of multicultural teaching environments, in addition to assessing their teaching self-efficacy. This is because self-efficacy beliefs are believed to be domain specific; individuals may have a firm and well-established sense of self-efficacy in one domain but not in another (Bandura, 1997). Teachers may evaluate or perceive of themselves as efficacious in a particular discipline and in teaching a specific group of students but less efficacious in teaching other groups of students (Tschannen-Moran et al., 1998). Hence, university instructors’ self-efficacy beliefs regarding teaching students from different cultural and ethnic backgrounds need to be examined in the context of post-secondary diversity.

### Multicultural Efficacy, Mental Well-Being, and Burnout

Similar to self-efficacy beliefs, multicultural efficacy has been examined in the context of teaching (e.g., Kang et al., 2019; Nadelson et al., 2012). While some evidence suggests that teachers’ self-efficacy and multicultural efficacy are distinct constructs (Mulder, 2010), to our knowledge, this association has yet to be explored among instructors in higher education. The few studies that have examined multicultural efficacy among university instructors have produced mixed findings. Vladimirschi (2012) examined multicultural efficacy of online university instructors and found participants were aware of cultural diversity and had a strong sense of multicultural efficacy with regard to the implementation of methods (e.g., “open communication”) that fostered learning and avoided conflict (p. 8). These instructors used various strategies in their instruction as well (e.g., modifying the content and methods of instruction to better address the needs of their diverse students, having flexibility with deadlines, fostering collaborations among diverse students, providing additional support to students with language proficiency issues, etc.). Lawson-Davenport (2014) measured multicultural efficacy of 30 university instructors teaching at four community college campuses in the United States. Unlike the findings of Vladimirschi (2012), it was found that instructors possessed low average levels of multicultural efficacy. Further, college instructors’ cultural awareness – acknowledging culture and cultural differences as well as taking into consideration the way in which such differences impact communication and collaboration (Murray & Bollinger, 2001) – had a positive association with and significantly predicted their multicultural efficacy (Lawson-Davenport, 2014). The small sample size employed in this study limited the generalizability of the findings to instructors of other institutions. Moreover, instructors’ low average levels of multicultural efficacy necessitate the need for measuring their mental well-being and burnout given that teaching in heterogeneous classrooms poses great challenges on educators (Chouari, 2016).

Teaching in multicultural contexts may involve some additional work for instructors, such as “reinvent[ing] syllabi, assessments, and general classroom delivery” (Bigatti et al., 2012, p. 79). Using a mixed-method approach, Jin and Schneider (2019) examined the perceptions of 261 faculty members toward international students in a U.S. university. The results indicated that some instructors found it challenging to evaluate the written work or oral presentations of international students, comprehend their spoken English, and involve them in activities conducted in the class (Jin & Schneider, 2019). Similarly, in a Canadian qualitative study, the language barrier of international students, additional efforts to edit their work, and plagiarism were among the challenges that professors faced (Heringer, 2021).

The challenging nature of instructing in multicultural settings should not undermine the richness that international students add to classrooms. Trice (2003) interviewed faculty members from four departments of architecture, mechanical engineering, materials science and engineering, and public health at a U.S. university about their perceptions of international students. Across all departments, faculty pointed to the importance of international perspective that these students provided. Faculty members at the engineering department noted that the presence of international students equip students for the multicultural dynamics of the workplace. Several faculty members in public health and architecture departments highlighted that international students add great insights to classrooms discussions and also enhance the learning experiences of local students. Faculty members across all departments also highlighted the academic excellence of international students, and those in engineering departments acknowledged the strong mathematical background of them. In this vein, one faculty noted that “[…] it really pushes the American students to get up to their level [in math]” (p. 393). Faculty members in architecture and public health departments noted that international students and, in particular, the students’ affiliation with their country of origin offered networking opportunities for partnership in research. Additionally, it created the opportunity for departments to formally connect with foreign organizations by introducing them “to high-level academic, corporate, and governmental leaders” (Trice, 2003, p. 393). Faculty members in these departments regarded the mere existence of international students effective in boosting the prestige and standing of their departments.

Other studies have also confirmed the positive contributions of international students. For example, Jin and Schneider (2019) found that faculty perceived these students to provide unique perspectives, demonstrate greater academic achievements, add globalized views to discussions and assignments, and bring linguistic diversity to the institution. Guo and Jamal (2007) discussed that the increase in student diversity brings about “an improvement in intergroup relations and campus climate, increased opportunities for accessing support and mentoring systems, opportunities for acquiring broader perspectives and viewpoints, and participating in complex discussions, all of which can contribute to increased learning” (p. 30).

The challenges of instructing in multicultural classrooms can be associated with development of diversity-related burnout defined as “the extent to which the teachers’ personal and professional well-being is negatively affected by the daily coping with a culturally heterogeneous student body” (Tatar & Horenczyk, 2003, p. 404). Research suggests that educators’ attitudes and beliefs toward diversity may place them at risk of developing this form of burnout. Researchers have examined the link between teachers’ attitudes and both general and diversity-related burnout (Dubbeld et al., 2019a). Strongly held assimilative attitudes toward diversity were associated with teachers’ high scores on both types of burnouts. The endorsement of multicultural attitudes was not linked to burnout (Dubbeld et al., 2019a). However, in another study, Dubbeld and colleagues (2019b) found the lowest levels of both general and diversity-related burnouts among teachers with multicultural attitudes (i.e., endorsement of multicultural ideology) and the perception of working in a pluralistic context. An increase in the diversity of students in post-secondary institutions may place instructors at risk of developing burnout. However, similar to self-efficacy beliefs, multicultural efficacy may act as a buffer against burnout. To help prevent university instructors from experiencing burnout, the relationships between stress and challenges involved with teaching in pluralistic settings needs further investigation.

### Current Study

The review of the literature indicates that most studies examining the link between self-efficacy beliefs and burnout have focused on school teachers (e.g., Capone et al., 2019; Weissenfels et al., 2021). While some studies have investigated burnout among instructors in higher education (e.g., El-Ibiary et al., 2017; Galindo-Domínguez & Pegalajar, 2020; Urbina-Garcia, 2020), little attention has been paid to the association of multicultural efficacy with burnout. The mental health status of school teachers and its contributing factors (e.g., age, gender, years of teaching experience) have been well documented (e.g., Desouky & Allam, 2017; Jones-Rincon & Howard, 2019). However, there are limited studies examining the potential link between multicultural efficacy and university instructors’ mental well-being. The purpose of the current study was to address this gap by (a) measuring the multicultural efficacy levels of university instructors, and (b) identifying whether multicultural efficacy remained as a significant predictor of mental well-being and burnout after controlling for demographics (e.g., age, gender, marital status), job-related characteristics (e.g., academic rank, years of teaching experience), colour-blind racial attitudes, and a sense of teaching self-efficacy in general. Following Mulder (2010) who suggested multicultural efficacy and teachers’ self-efficacy were distinct constructs, we hypothesized that multicultural efficacy would remain a significant predictor of (a) burnout, and (b) mental well-being, after accounting for demographics, job-related characteristics, a sense of teaching efficacy, and colour-blind racial attitudes.

## Method

### Procedure

Questionnaires were administered via Qualtrics, an online surveying software. Initial ethical approval was obtained from a medium-sized Canadian University's Research Ethics Board (File# 2018-122). The data collection took place in 2018. It is important to note that this study has been conducted prior to the COVID-19 outbreak, which led to changes in universities (e.g., the replacement of online distance education for the traditional face-to-face mode of learning; Nichols et al., 2022). To maximize recruitment efforts, the researchers gained permission from the Ethics Board at the Universities of Regina, Saskatchewan, Alberta, and British Colombia to contact faculty and instructors via email. After providing consent, participants completed a questionnaire measuring demographics, multicultural efficacy, teachers’ sense of efficacy, colour-blind racial attitudes, burnout, and mental well-being in a randomized order.

### Participants

Participants were recruited from faculty and sessional instructors at the University of Regina and its federated colleges (*n* = 40), University of Saskatchewan (*n* = 44), University of Alberta (*n* = 33), and University of British Columbia (*n* = 21). Some participants (*n* = 20) were from universities other than those mentioned above or chose not to state their affiliated universities. With the exception of emeritus faculty, participants regardless of their status (e.g., on sabbatical, maternity, study, research, and medical leave) were eligible to participate in this study. University instructors who participated in the current study belonged to a broad cross-section of academic units corresponding to sciences and engineering, social sciences, humanities, and health/life sciences. The initial sample consisted of 164 participants. However, six cases were identified as univariate outliers with z-scores exceeding ±3.29 standard deviation units from the mean (*p* < .001, two-tailed test; Tabachnik & Fidell, 2013). These outliers were removed from the dataset and not included in further analyses. No multivariate outliers were identified. The final sample comprised of 158 participants (*M*_age_ = 48.51, *SD* = 10.77; *Range* 26-78; *n* = 89 females). A summary of pertinent demographics can be found in Table 1.

**Table 1.** Summary of Demographics.

Age: *M* (*SD*)	48.51 (10.77)
Years of teaching experience	16.11 (10.86)
Percentage of diverse students	38.82 (23.69)
Gender: n (%)	
Female	89 (56.3)
Male	67 (42.4)
Non-binary/non-conforming	2 (1.3)
Native English speakers	122 (77.2)
Ethnicity	
White/European descent	128 (81.0)
Asian	12 (7.6)
Middle Eastern	5 (3.2)
Aboriginal	3 (1.9)
Hispanic	2 (1.3)
African	1 (.6)
Other	7 (4.4)
Marital status	
Married	113 (71.5)
Single/Never married	13 (8.2)
Separated/Divorced	10 (6.3)
Missing	22 (13.9)
Academic rank	
Professor	50 (31.6)
Associate professor	44 (27.8)
Assistant professor	27 (17.1)
Instructor/Lecturer/Other	27 (17.1)
Sessional	10 (6.3)
Academic degree	
Doctorate	130 (82.3)
Master’s	23 (14.6)
Bachelor’s	5 (3.2)
University	
Regina	40 (25.3)
Saskatchewan	44 (27.8)
Alberta	33 (20.9)
British Colombia	21 (13.3)
Other	20 (12.7)

### Measures

#### Demographics and Job-Related Characteristics

Participants were asked a series of questions regarding gender, age, ethnicity, marital status, years of teaching experience, mode of teaching, academic rank, academic degree, university, and department.

#### Multicultural Efficacy Scale

The 20-item Efficacy subscale of the Multicultural Efficacy Scale (MES; Guyton & Wesche, 2005) was used to measure university instructors’ multicultural efficacy levels. Participants are asked to evaluate their capabilities with respect to various statements (e.g., “*I can get students from diverse groups to work together*”) on a 4-point Likert scale, ranging from 1 (“*I do not believe I could do this very well”*) to 4 (“*I am quite confident that this would be easy for me to do”*). On this subscale, scores can range from 20-54 (low), 55-66 (average), and 67-80 (high). Guyton and Wesche (2005) have shown that the Efficacy subscale possessed internal reliability of .93. In the current study, the internal reliability of the Efficacy subscale was excellent (α = .94).

#### Teachers’ Sense of Efficacy Scale

The short version of the Teachers’ Sense of Efficacy Scale (TSES; Tschannen-Moran & Hoy, 2001) was administered to measure participants’ sense of efficacy in teaching. Participants are asked to respond to 12 items (e.g., “*To what extent can you use a variety of assessment strategies?*”) on a 9-point Likert scale, varying from 1 (“*Nothing”*) to 9 (“*A Great deal”*). Higher scores represent stronger levels of teaching efficacy beliefs. Given that the scale has been developed to assess prospective and in-service school teachers’ self-efficacy levels in teaching, some of the items were slightly modified (e.g., school work was replaced with course work), and a post-secondary terminology was utilized to better reflect the higher education context (Fives & Looney, 2009). Tschannen-Moran and Hoy (2001) have obtained excellent reliability (α = .90) with use of full-scale items. In the current study, the total scores were used for analyses and good internal reliability (α = .87).

#### Colour-Blind Racial Attitudes Scale

The 20-item Colour-Blind Racial Attitude Scale (CoBRAS; Neville et al., 2000) was used to assess colour-blind racial beliefs. In particular, the CoBRAS measures the extent to which individuals deny, distort, and minimize that racism and issues related to race exist. In this study, the CoBRAS was adapted for use in Canada by taking out the references to the “U.S.” and “America”. Participants are asked to rate their agreement with various statements (e.g., “*Racism may have been a problem in the past, but it is not an important problem today*”) on a 5-point Likert scale, varying from 1 (*“Strongly Disagree”*) to 5 (*“Strongly Agree”*). Scores on this instrument ranged from 20 to 100, with higher scores reflective of greater levels of denial that institutional racism exists. Neville et al. (2000) have reported good internal reliability for the full-scale items, ranging from .86 to .91. In the current study, the full-scale had excellent internal reliability of α = .92.

#### Maslach Burnout Inventory-Educators Survey

The Maslach Burnout Inventory-Educators Survey (MBI-ES; Maslach et al., 1996) is a measure of instructors’ burnout levels. The scale consists of Emotional Exhaustion (EE), Depersonalization (DP), and Personal Accomplishment (PA). Participants are asked to rate their agreement, on a 7-point Likert-type scale, varying from 0 (“*Never”*) to 6 (*“Every day”*), with 22 different statements. Higher levels of burnout are reflected by higher scores on the EE and DP subscales, and lower scores on the PA component. Since the three subscales are designed to be orthogonal, an average score was calculated for each dimension of burnout (Maslach et al., 1997). The EE, DP, and PA subscales have shown to possess excellent internal reliability in previous studies (.90, .76, and .76, respectively, Iwanicki & Schwab, 1981). In this study, internal reliability of the EE, DP, and PA subscales were α = .91, .68, and .82, respectively.

#### The Warwick-Edinburgh Mental Well Being Scale

The Warwick-Edinburgh Mental Well-Being Scale (WEMWBS; Tennant et al., 2007) was used to assess positive mental well-being. The WEMWBS is comprised of 14 items (e.g., “*I have been dealing with problems well*”) rated on a 5-point Likert-type scale, varying from 1 (“*None of the time”*) to 5 (*“All of the time”*). The scores range from 14 to 70, and higher scores are reflective of higher mental well-being. In a previous study, researchers found the WEMWB to possess excellent internal consistency of α = .94 (Marmara et al., 2022). In this study, the scale demonstrated an excellent internal reliability of α = .92.

### Data analysis Plan

#### Power Analysis

G*Power 3.1.9.4 was utilized to calculate the sample size needed for this study. For a hierarchical regression analysis with a medium effect size of f = .15, alpha = .05, and power = .95, three tested predictors, and seven total predictors, the estimated number of participants needed for this study was *N* = 119. We sought to slightly over-sample in anticipation of missing data or removal of outliers.

Prior to conducting the analyses, data were checked for outliers. Skewness and kurtosis for the key measures of interest as well as residual plots from all regression analyses were assessed to determine appropriateness of the analytic approach. Acceptable ranges for skewness were set at −1 to 1 and for kurtosis were from −2 to 2 (George & Mallery, 2010). Additionally, residual plots were examined to check for homoscedasticity, linearity, and normality in the data. Any violations in the above-mentioned metrics resulted in data transformation. Multicollinearity was also examined for key variables in each regression model, using Tolerance (i.e., values above 0.2; Menard, 1995) and Variance Inflation Factor (i.e., values less than 10; Myers, 1990).

### Hypotheses

We hypothesized multicultural efficacy would negatively associate with Emotional Exhaustion and Depersonalization facets of burnout but positively associated with Personal Accomplishment dimension of burnout and mental well-being. To validate these hypotheses, we conducted four hierarchical linear regressions with the three components of burnout and mental well-being as dependent variables. To control for the impact of demographics and job-related characteristics, marital status, years of teaching experience, academic rank, and gender were entered in the first step of the regressions. Given that Age and teaching experience were highly correlated, *r*(155) = .78, *p* < .001, teaching experience was used in the regression model due to its relevance to the present study. Colour-blind racial attitudes along with teaching self-efficacy were entered in the second step. Multicultural efficacy was entered in the third step of each model.

## Results

### Preliminary Data Check

A summary of the mean and standard deviations for all measures are reported in Table 2. University instructors possessed average levels of multicultural efficacy. They also reported moderate levels of Emotional Exhaustion, low levels of Depersonalization, and approximately high levels of Personal Accomplishment.

**Table 2.** Descriptive Statistics.

Variable	*M*	*SD*	Skewness	Kurtosis	Potential Range	Observed Range
MES	63.66	10.61	−.68	.40	20–80	27–80
TSES	87.21	10.83	−.52	.32	12–108	51–108
CoBRAS	39.29	12.67	.59	−.30	20–100	20–78
MBI_EE	2.63	1.33	.14	−.68	0–6	.00–5.67
MBI_DP	1.00	.87	1.09	.60	0–6	.00–3.40
MBI_PA	4.53	.89	−.72	.26	0–6	1.75–6
WEMWBS	52.52	7.22	−.08	.16	14–70	32–70

*Note*. ME = Multicultural Efficacy; TSES = Teachers’ Sense of Efficacy Scale; CoBRAS = Colour-Blind Racial Attitudes Scale; MBI_EE = Maslach Burnout-Emotional Exhaustion; MBI_DP = Maslach Burnout-Depersonalization; MBI_PA = Maslach Burnout-Personal Accomplishment; WEMWBS = Warwick-Edinburgh Mental Well-Being Scale.

Participants’ scores on the measures of interest were within acceptable ranges for skewness and kurtosis (see Table 2). The Depersonalization facet of burnout (MBI_DP) was slightly skewed (1.09). We used Log transformation to transform the data on this variable and used the transformed variable in the subsequent analyses. All other variables met the assumptions of normality, linearity, and homoscedasticity. No multicollinearity was evident in the data. A correlational analysis was conducted to examine the associations between the predictor variables (See Table 3).

**Table 3.** Correlations Between Study Variables.

	1	2	3	4	5	6	7	8	9	10
1. Marital status										
2. Academic rank	−.07									
3. Years of teaching experience	.11	−.54***								
4. Gender	−.20*	.21**	−.23**							
5. ME	−.15	.04	.02	.23**						
6. TSES	.05	.08	.04	.17*	.45***					
7. CoBRAS	.15	.01	.13	−.24**	−.39***	−.13				
8. MBI_EE	−.28***	−.04	−.09	.20*	.07	−.10	−.27***			
9. MBI_DP	−.06	−.04	−.11	−.01	−.19*	−.31***	−.05	.46***		
10. MBI_PA	.03	.06	.08	.10	.40***	.43***	−.08	−.21**	−.20*	
11. WEMWBS	.08	.03	.04	.02	.20*	.29***	.12	−.57***	−.30***	.65***

*Note*. ME = Multicultural Efficacy; TSES = Teachers’ Sense of Efficacy Scale; CoBRAS = Colour-Blind Racial Attitudes Scale; MBI_EE = Maslach Burnout-Emotional Exhaustion; MBI_DP = Maslach Burnout-Depersonalization; MBI_PA = Maslach Burnout-Personal Accomplishment; WEMWBS = Warwick-Edinburgh Mental Well-Being Scale.

*p < .05, **p < .01, ***p < .001.

### Predicting Burnout and Mental Well-Being

For the Emotional Exhaustion facet of burnout, the overall hierarchical regression model was statistically significant, *F*(7, 123) = 2.36, *p* < .03, and accounted for 11.8% of the variance (Table 4). In the final regression equation, only marital status (*β* = −.23, *p* < .02) was found as a significant predictor of Emotional Exhaustion. Compared to married instructors, those who were single, divorced, or separated possessed higher levels of Emotional Exhaustion.

**Table 4.** Hierarchical Regression Predictors of MBI_EE.

Step/Variable	B	SE B	β	*R*^*2*^ Increase	*F* for *Δ* *R*^*2*^
Step 1				.08	2.82*
Marital status	−.87	.31	−.25**		
Academic rank	−.07	.28	−.03		
Teaching experience	−.01	.01	−.05		
Gender	.25	.24	.09		
Step 2				.03	2.35
Marital status	−.78	.31	−.22*		
Academic rank	−.00	.28	−.00		
Teaching experience	−.00	.01	−.01		
Gender	.22	.24	.08		
TSES	−.02	.01	−.13		
CoBRAS	−.02	.01	−.16		
Step 3				.00	.37
Marital status	−.81	.32	−.23*		
Academic rank	.01	.28	.00		
Teaching experience	−.00	.01	−.01		
Gender	.23	.24	.09		
TSES	−.01	.01	−.11		
CoBRAS	−.02	.01	−.18		
ME	−.01	.01	−.06		

*Note*. ME = Multicultural Efficacy; TSES = Teachers’ Sense of Efficacy Scale; CoBRAS = Colour-Blind Racial Attitudes Scale; MBI_EE = Maslach Burnout-Emotional Exhaustion.

*Note.* Final *R*^*2*^ = .118; *p < .05, **p < .01, ***p < .001.

For the Depersonalization dimension of burnout, the overall model was trending towards statistical significant, *F*(7, 108) = 2.15, *p* = .045 and accounted for 12.2% of the variance. In the final regression equation, teaching self-efficacy was a significant predictor of this facet of burnout. Higher levels of teaching self-efficacy were associated with lower levels of Depersonalization among university instructors, (*β* = −.23, *p* < .03 (Table 5).

**Table 5.** Hierarchical Regression Predictors of MBI_DP_ Transformed.

Step/Variable	B	SE B	β	*R*^*2*^ Increase	*F* for *Δ* *R*^*2*^
Step 1				.03	.93
Marital status	−.11	.10	−.12		
Academic rank	−.08	.08	−.11		
Teaching experience	−.01	.00	−.15		
Gender	−.05	.07	−.07		
Step 2				.07	4.43
Marital status	−.08	.09	−.08		
Academic rank	−.05	.08	.07		
Teaching experience	−.00	.00	−.13		
Gender	−.02	.07	−.03		
TSES	−.01	.00	−.28**		
CoBRAS	−.00	.00	−.05		
Step 3				.02	2.08
Marital status	−.10	.09	−.10		
Academic rank	−.05	.08	−.06		
Teaching experience	−.00	.00	−.11		
Gender	−.01	.07	−.01		
TSES	−.01	.00	−.23*		
CoBRAS	−.00	.00	−.10		
ME	−.01	.00	−.16		

*Note*. ME = Multicultural Efficacy; TSES = Teachers’ Sense of Efficacy Scale; CoBRAS = Colour-Blind Racial Attitudes Scale; MBI_DP = Maslach Burnout-Depersonalization.

*Note.* Final *R*^*2*^ = .122; *p < .05, **p < .01, ***p < .001.

For the Personal Accomplishment aspect of burnout, the overall model was statistically significant, *F*(7, 123) = 6.15, *p* < .001, and accounted for 25.9% of the variance (Table 6). Even after accounting for the impact of teaching self-efficacy, multicultural efficacy remained a significant predictor of Personal Accomplishment. In the final regression equation, teaching self-efficacy (*β* = .29, *p* < .01) and multicultural efficacy (*β* = .31, *p* < .01) were identified as significant predictors of Personal Accomplishment. Therefore, high levels of teaching self-efficacy and multicultural efficacy were associated with high levels of Personal Accomplishment over and above variance accounted for by demographics, job-related characteristics, and colour-blind racial attitudes.

**Table 6.** Hierarchical Regression Predictors of MBI_PA.

Step/Variable	B	SE B	β	*R*^*2*^ Increase	*F* for *Δ* *R*^*2*^
Step 1				.03	.91
Marital status	.11	.22	.05		
Academic rank	.25	.20	.14		
Teaching experience	.01	.01	.15		
Gender	.18	.17	.10		
Step 2				.17	12.93***
Marital status	.04	.21	.01		
Academic rank	.17	.18	.09		
Teaching experience	.01	.01	.10		
Gender	−.00	.16	−.00		
TSES	.03	.01	.41***		
CoBRAS	−.01	.01	−.08		
Step 3				.06	10.56**
Marital status	.14	.20	.06		
Academic rank	.13	.18	.07		
Teaching experience	.01	.01	.06		
Gender	−.05	.16	−.03		
TSES	.02	.01	.29**		
CoBRAS	.00	.01	.02		
ME	.03	.01	.31**		

*Note*. ME = Multicultural Efficacy; TSES = Teachers’ Sense of Efficacy Scale; CoBRAS = Colour-Blind Racial Attitudes Scale; MBI_PA = Maslach Burnout-Personal Accomplishment.

*Note.* Final *R*^*2*^ = .259; *p < .05, **p < .01, ***p < .001.

For mental well-being, the overall model was statistically significant, *F*(7, 123) = 2.69, *p* < .02, and accounted for 13.3% of the variance (Table 7). Even after controlling for teaching self-efficacy, multicultural efficacy was identified as a significant predictor of mental well-being. In the final regression equation, teaching self- efficacy (*β* = .23, *p* < .02) and multicultural efficacy (*β* = .22, *p* < .03) were identified as significant predictors of mental well-being.

**Table 7.** Hierarchical Regression Predictors of WEMEBS.

Step/Variable	B	SE B	β	*R*^*2*^ Increase	*F* for *Δ* *R*^*2*^
Step 1				.01	.19
Marital status	1.37	1.91	.07		
Academic rank	−.66	1.72	−.04		
Teaching experience	−.00	.08	−.00		
Gender	.66	1.45	.04		
Step 2				.09	6.34**
Marital status	.64	1.85	.03		
Academic rank	−1.30	1.66	−.08		
Teaching experience	−.04	.07	−.06		
Gender	−.14	1.45	−.01		
TSES	.22	.06	.32***		
CoBRAS	.04	.06	.06		
Step 3				.03	4.87*
Marital status	1.30	1.85	.06		
Academic rank	−1.54	1.64	−.10		
Teaching experience	−.06	.07	−.08		
Gender	−.40	1.44	−.03		
TSES	.16	.07	.23*		
CoBRAS	.07	.06	.12		
MES	.16	.07	.22*		

*Note*. ME = Multicultural Efficacy; TSES = Teachers’ Sense of Efficacy Scale; CoBRAS = Colour-Blind Racial Attitudes Scale; WEMWBS = Warwick-Edinburgh Mental Well-Being Scale.

*Note.* Final *R*^*2*^ = .133; *p < .05, **p < .01, ***p < .001.

## Discussion

To our knowledge, this study was the first to examine university instructors’ burnout and mental well-being as a function of their multicultural efficacy beliefs. We explored the association of multicultural efficacy with burnout and mental well-being after accounting for demographics, job-related characteristics, colour-blind racial attitudes, and teaching self-efficacy. The increase in ethnically and culturally diverse students require instructors to possess multicultural efficacy and provide adaptive teaching methods to meet the needs of their diverse students. Instructors who lack such qualities may not perceive themselves as efficacious instructors and may fail to overcome the challenges of multicultural classrooms. This may exacerbate the burnout many instructors already report and potentially reduce their mental well-being, which, in turn, may result in lower occupational effectiveness and satisfaction, and increase turnover intentions.

University instructors in our sample possessed average levels of multicultural efficacy. This was consistent with previous studies of prospective teachers (Nadelson et al., 2012). However, such comparisons are imperfect as prospective teachers are a different population than experienced post-secondary instructors. Prospective teachers do not have substantial experience instructing in ecologically valid classrooms as yet, and hence, their perceptions of self-efficacy may change when they gain more teaching experience.

Inconsistent with our hypothesis, multicultural efficacy was not identified as a significant predictor of Emotional Exhaustion. This finding was counter to Chahar Mahali and Sevigny (2022) who reported that prospective teachers with higher levels of culturally responsive teaching self-efficacy possessed lower levels of Emotional Exhaustion. Similarly, El-Sayed et al. (2014) found a negative link between occupational stress and self-efficacy among a sample of nursing faculty members. The nonsignificant association between multicultural efficacy and Emotional Exhaustion in the context of this study may be partially explained by instructors’ academic rank and length of teaching experience. Descriptive analysis illustrated that, on average, instructors had 16 years of teaching experience, and 94 of them had high designation (i.e., Professor and Associate Professor). Experienced university instructors might be better able to navigate the challenges of instructing in multicultural settings than novices. We found marital status was significantly associated with this facet of burnout. Compared to their married counterparts, university instructors who were single, divorced, or separated reported higher levels of Emotional Exhaustion. This was consistent with previous studies reporting lower levels of Emotional Exhaustion among married and cohabitating teachers compared with those in single or widowed, divorced, and separated categories (Wang et al., 2015). One potential explanation for the lower levels of Emotional Exhaustion among married university instructors in the current sample may be attributed to their social support from their partners, and therefore, they may be better equipped to cope with their job demands (Wang et al., 2015).

While multicultural efficacy was not a significant predictor of Depersonalization, teaching self-efficacy was a significant predictor of this component of burnout. UniverAsity instructors with higher levels of teaching self-efficacy reported lower levels of Depersonalization. This finding corroborated the results of previous studies on the negative association between depersonalization and teaching self-efficacy among school teachers (Capone & Petrillo, 2020). No other variables were identified as a significant predictor of Depersonalization. It is possible that Depersonalization develops in response to other factors such as unfairness within the workplace (Maslach et al., 2001). Smith et al. (2007) reported a negative link between university instructors’ perceptions of having dedicated and competent students and Depersonalization. These authors suggested that instructors may use detachment as a way to cope with students who are incompetent and lacking dedication. Another possibility is that those who score high on Depersonalization may perceive their students as having such characteristics (Smith et al., 2007).

The results of our study revealed a negative relationship between colour-blind racial attitudes and multicultural efficacy. This was similar to Aragón et al. (2017) who found instructors’ strong endorsement of colourblind racial ideology was related to lower implementation of inclusive teaching practices. Aragón et al. (2017) defined colourblind racial ideology “with an emphasis on sameness, equal treatment for all, and the idea that all have equal opportunities” (p. 206). Colour-blind racial attitudes are characterized by minimizing, distorting, and denying the existence of institutional and ideological racism as well as not being cognizant of them (Neville et al., 2000). To better address the needs of diverse students, educators should explore their beliefs towards diversity and realize the impact of their attitudes on the way they interact with such students (Giambo & Szecsi, 2007).

Our results also revealed that multicultural efficacy remained a significant predictor of Personal Accomplishment and mental well-being even after controlling for demographics, job-related characteristics, colour-blind racial attitudes, and teaching self-efficacy. These findings were in line with the results of Huang et al. (2019) in which school teachers’ self-efficacy beliefs were found to be inversely linked to anxiety and depression. Similarly, Matos et al. (2022) found self-efficacy was a positive predictor of Personal Accomplishment among a sample of Brazilian university lecturers. The results of our study indicated that both teaching self-efficacy and multicultural efficacy were positive and significant predictors of Personal Accomplishment and mental well-being. Even after accounting for the variance accounted for by teaching self-efficacy, multicultural efficacy was identified as a significant predictor of Personal Accomplishment and mental well-being. Accordingly, the results of this study indicated that multicultural efficacy and teaching self-efficacy, albeit related, are different constructs.

The predictive role of multicultural efficacy above and beyond that of the teaching self-efficacy further suggests that that self-efficacy beliefs are domain-specific. Therefore, merely having generalized teaching self-efficacy beliefs is not adequate for effective instruction in heterogenous contexts. The multicultural and challenging nature of today’s diverse classrooms require university instructors to possess multicultural efficacy beliefs to have more salient perceptions of Personal Accomplishment and stronger mental well-being. This, in turn, may lower instructors’ likelihood of developing burnout or experiencing occupational dissatisfaction. Moreover, instructors’ multicultural efficacy beliefs may be linked to positive student outcomes in diverse classrooms, as evidence points to the association of teachers’ self-efficacy beliefs with student achievement and motivation (Mojavezi & Tamiz, 2012). Results of the current study suggest to gain a deeper appreciation of the correlates of burnout and well-being among educators, a more nuanced assessment of domain specific self-efficacy beliefs is needed.

Effective instruction in multicultural environments requires instructors be prepared to work with a heterogeneous body of students and acknowledge their differences. The findings of the present study indicated that multicultural efficacy was negatively linked to colour-blind racial attitudes and Depersonalization aspect of burnout. Furthermore, multicultural efficacy was shown to be associated with Personal Accomplishment and mental well-being. These results can inform the development of training opportunities and diversity-related workshops to enhance instructors’ awareness of diversity, social justice issues, and multicultural efficacy.

### Limitations

Several limitations pave the way for future research. First, this was a cross-sectional study and accordingly, no conclusions regarding causal links or directionality between variables can be drawn. Second, we used self-reported measures and note what instructors reported may be subject to social desirability biases and may not fully reflect the true nature of their beliefs, attitudes, well-being, and burnout. Third, university instructors were recruited mainly from four Canadian universities and the results cannot be generalized to instructors in other institutions due to the cross-sectional design of the study and small sample size. Fourth, the majority of participants were of White European descent, and the pattern of results may differ among ethnic minority instructors. Fifth, the CoBRAS is a 6-point Likert scale, with scores typically ranging from 1 to 6. However, in the current study, the scores ranged from 1 to 5. Sixth, the Depersonalization facet of burnout possessed a low reliability score. However, in their meta-analysis, Wheeler et al., (2011) reported that the range of the coefficient alpha was from .50 to .91 for this aspect of burnout. Finally, we did not focus on systematic discrimination or marginalization of university faculty as an engine of burnout. The examination of this potential link is critical, as scholars have indicated that minority faculty experience marginalization and exclusion as a result of “the White elite culture [that] shapes and controls the power structure in the academy” (Orelus, 2020, p. 128).

### Future Directions

Since social desirability may impact responses on self-administered measures, objective methods (e.g., recording interactions in classrooms) should be implemented to verify the validity of participants’ responses. Mixed-methods of inquiry (i.e., quantitative and qualitative) should also be utilized to examine the challenges of multicultural classrooms and gain a deeper understanding of instructors’ self-efficacy beliefs with regard to instruction in such settings. Future studies should examine the effectiveness of diversity-related workshops in enhancing multicultural efficacy as well as cultural awareness of instructors. In addition, the association of instructors’ multicultural efficacy beliefs and attitudes toward diversity (e.g., assimilationist, colour-blind, or pluralistic) with diverse students’ academic performance should be studied. Finally, researchers should also study whether multicultural efficacy beliefs are linked to turnover intentions and job satisfaction.

### Conclusion

The enrollment of students from diverse backgrounds in Canadian universities is increasing. Therefore, teaching in multicultural contexts requires university instructors to be cognizant of their diversity-related attitudes and possess multicultural efficacy beliefs. It is important that university instructors recognize diverse racial, cultural, and ethnic profiles of their students to adapt their methods of instruction and embrace diversity within their classrooms. This, in turn, may enhance students’ sense of belongingness and engagement. Instructors who perceive themselves as multiculturally efficacious have positive evaluations of their personal accomplishments and higher mental well-being, which, in turn, fosters meaningful relationships with those they interact with. Such instructors are more likely to overcome the challenges of multicultural classrooms and address the needs of their diverse students.

## Data Availability

The dataset used and/or analyzed during the current study will be available from the corresponding author on reasonable request. *
